# 2156: A confounder assessment of patient frailty in the relationship between antidiabetic medication and heart failure

**DOI:** 10.1017/cts.2017.97

**Published:** 2018-05-10

**Authors:** Caroline Presley, Marie Griffin, Jea Young Min, Robert Greevy, Christianne Roumie

**Affiliations:** Vanderbilt University, Nashville, TN, USA

## Abstract

OBJECTIVES/SPECIFIC AIMS: This study is part of a parent grant evaluating antidiabetic medications and risk for heart failure in an observational cohort of Veterans with type 2 diabetes (T2DM). Confounding by indication remains a concern in many observational studies of medications because difficult to measure confounders such as frailty may influence prescribing of different medications based on patient characteristics. Frailty is a multidimensional syndrome of loss of reserves (energy, physical ability, cognition, health) that gives rise to vulnerability to adverse outcomes. The objective of this study is to determine if frailty is a potential confounder in Veterans with T2DM, that is, independently associated with exposure to a specific antidiabetic medications and hospitalization for decompensated heart failure. METHODS/STUDY POPULATION: We conducted a cross-sectional study of patients with diabetes who were hospitalized within the Veterans Health Administration (VHA) Tennessee Valley Healthcare System from 2002 to 2012. Inclusion criteria were: age 18 years or older, receive regular VHA care (prescription fill or visit at least once every 180 d), a diagnosis of T2DM. A probability sample of HF and non-HF hospitalizations was collected. HF hospitalizations were selected on the basis of meeting either a primary diagnosis code (ICD-9) and/or disease related group (DRG) code for HF. For each hospitalization using a standardized chart abstraction tool, data was abstracted on: antidiabetic medication(s), patient frailty status, and reason for hospitalization (HF or non-HF). Antidiabetic medication regimens were categorized as follows: no medication treatment, metformin alone, sulfonylurea alone, insulin alone, insulin and one oral agent, and all other regimens. Patient frailty status was measured using a modified version of the Canadian Health and Aging frailty index (FI), which generates a score (range 0–1) by dividing the number of deficits present by the number of deficits measured. Established categories for FI scores are: non frail ≤0.10, vulnerable 0.10–0.21, frail 0.22–0.45, and very frail >0.45. Patient frailty status at the time of hospitalization was used as a surrogate for patient frailty at the time of prescription of antidiabetic medication; this is a limitation of this approach. Hospitalizations were classified as HF hospitalizations if 2 major or 1 major and 2 minor Framingham criteria were present. FI was compared across antidiabetic medication regimen categories and hospitalization type using analysis of variance (ANOVA) and Student *t*-test, respectively. RESULTS/ANTICIPATED RESULTS: Of the 500 hospitalizations reviewed, 430 patients had confirmed diabetes diagnosis, adequate data to calculate FI scores, and were included in this analysis. Patients were on average 66.9 (10.9) years old; 99% male and 75% were White. Overall, 268 patients (62.3%) were categorized as frail or very frail. The mean FI score was 0.23 (SD 0.07). FI scores were highest in patients receiving insulin alone (mean 0.26) compared with patients receiving metformin alone (mean 0.22), sulfonylurea alone (mean 0.23), or no medication (mean 0.22). The lowest mean frailty score was seen in patients taking all other drug combinations, 0.19. The differences across these patient groups were statistically significant with *p*<0.01. Further, 75% of patients on insulin alone were frail or very frail compared with 68% on sulfonylurea alone, 58% on metformin alone, and 58% on no medication. Framingham criteria for acute HF were present for 318 of 430 patients (74.0%). FI scores were higher in patients hospitalized for HF compared with non-HF hospitalizations (mean 0.24 vs. 0.21, *p*<0.01). A higher proportion of patients hospitalized for HF were classified as frail or very frail compared with those hospitalized for non-HF diagnosis (66.4% vs. 50.9%, *p*<0.01). DISCUSSION/SIGNIFICANCE OF IMPACT: This study demonstrates that certain antidiabetic medications are associated with patient frailty. In addition, those patients admitted for HF have higher FI scores than those admitted for non-HF diagnoses. Further investigation is planned to assess the degree to which frailty is captured by traditional covariates used in observational studies.

